# A validation of the Postpartum Specific Anxiety Scale 12-item research short-form for use during global crises with five translations

**DOI:** 10.1186/s12884-021-03597-9

**Published:** 2021-02-08

**Authors:** Sergio A. Silverio, Siân M. Davies, Paul Christiansen, Marta E. Aparicio-García, Alessandra Bramante, Ping Chen, Natalia Costas-Ramón, Carolina de Weerth, Anna M. Della Vedova, Lilliam Infante Gil, Hellen Lustermans, Jaqueline Wendland, Jihong Xu, Jason C. G. Halford, Joanne A. Harrold, Victoria Fallon

**Affiliations:** 1 Department of Women & Children’s Health, School of Life Course Sciences, King’s College London, London, UK; 2 Department of Psychology, Institute of Population Health, University of Liverpool, Liverpool, UK; 3 Elizabeth Garrett Anderson Institute for Women’s Health, Faculty of Population Health Sciences, University College London, London, UK; 4 School of Psychology, Faculty of Health, Liverpool John Moores University, Liverpool, UK; 5 Division of Psychology and Mental Health, School of Health Sciences, The University of Manchester, Manchester, UK; 6 Departamento de Psicología Social, Psicología del Trabajo y Psicología Diferencial, Facultad de Psicología, Universidad Complutense de Madrid, Madrid, Spain; 7 Humanitas San Pio X, Milan, Italy; 8 Collaborative Innovation Center of Assessment for Basic Education Quality, Beijing Normal University, Beijing, China; 9 Department of Cognitive Neuroscience, Donders Institute for Brain, Cognition and Behaviour, Radboud University Medical Center, Nijmegen, The Netherlands; 10 Dipartimento di Scienze Cliniche e Sperimentali, Università degli Studi di Brescia, Brescia, Italy; 11 Laboratoire Psychopathologie et Processus de Santé, Institut de Psychologie, Université de Paris, Paris, France; 12 National Research Institute for Family Planning, Beijing, China; 13 School of Psychology, Faculty of Medicine and Health, University of Leeds, Leeds, UK

**Keywords:** Anxiety, Maternal mental health, Psychometric assessment, Postpartum

## Abstract

**Background:**

Global crises inevitably increase levels of anxiety in postpartum populations. Effective and efficient measurement is therefore essential. This study aimed to create a 12-item research short form of the 51-item Postpartum Specific Anxiety Scale [PSAS] and validate it for use in rapid response research at a time of global crises [PSAS-RSF-C]. We also present the same 12-items, in five other languages (Italian, French, Chinese, Spanish, Dutch) to increase global accessibility of a psychometric tool to assess maternal mental health.

**Methods:**

Twelve items from the PSAS were selected on the basis of a review of their factor loadings. An on-line sample of UK mothers (*N* = 710) of infants up to 12 weeks old completed the PSAS-RSF-C during COVID-19 ‘lockdown’.

**Results:**

Principal component analyses on a randomly split sample (*n* = 344) revealed four factors, identical in nature to the original PSAS, which in combination explained 75% of the total variance. Confirmatory factor analyses (*n* = 366) demonstrated the four-factor model fit the data well. Reliability of the overall scale and of the underlying factors in both samples proved excellent.

**Conclusions:**

Findings suggest the PSAS-RSF-C may prove useful as a clinical screening tool and is the first postpartum-specific psychometric scale to be validated during the COVID-19 pandemic. This offers psychometrically sound assessment of postpartum anxiety. By increasing the accessibility of the PSAS, we aim to enable researchers the opportunity to measure maternal anxiety, rapidly, at times of global crisis.

## Background

### The COVID-19 global pandemic context

The Coronavirus [SARS-CoV-2] or COVID-19 pandemic poses a devastating risk to the health of the global population. Amongst those thought to be most vulnerable are pregnant women and newborn infants, although guidance rapidly changed to state that pregnant women are no more vulnerable than the general population [1]. Although the growing body of evidence remains conflicting about the size of the risk to these populations, perinatal deaths have been reported [2]. This makes the perinatal period a time of increased vulnerability [3]. Whilst COVID-19 poses a serious physical health risk to those who contract the virus, there is evidence for it also affecting mental health outcomes [4, 5]. Poor mental health in relation to COVID-19 has been associated with various Government mandated restrictions, which have been enforced in an attempt to slow the spread of the virus. These include ‘quarantine’ (the enforced isolation of persons with or suspected of having the virus) [6]; ‘social distancing’ (the physical separation of persons outside of those in one’s family) [7]; ‘lockdown’ (the closure of public venues and banning of non-essential travel) [8]; and ‘shielding’ (where the most vulnerable – including pregnant women – are advised to remain at home and leave under no circumstances) [1]. Given the expected effect of the COVID-19 pandemic on mental health coupled with pregnant women and newborn infants being labelled as vulnerable groups [1], it is important to assess and understand the mental health effects in perinatal women [9]. During normal circumstances, approximately 20% of all women who give birth are thought to experience mental health problems [10]. The global pandemic is set to pose *“unprecedented challenges that can significantly impact on women’s mental health”* during the perinatal period [3], hence potentially driving these numbers even higher.

### Postpartum anxiety

In 2014, the National Institute of Health and Care Excellence [NICE] requested attention to the under-detection of postpartum anxiety in recognition of the significant burden it poses [11]. Postpartum anxiety is associated with many negative maternal and infant outcomes including reduced breastfeeding [12], reduced maternal sensitivity [13], impaired bonding [14], difficult infant temperament [15], atypical neurodevelopment [16], and child emotional and behavioural problems [17]. However, general measures of anxiety are relied upon in a large majority of studies examining postpartum anxiety, but are psychometrically problematic [11, 18].

The Postpartum Specific Anxiety Scale [PSAS] examines the frequency of maternal and infant focused anxieties experienced by women across the first year of their infants’ life [19]. The 51-item measure assesses four domains of anxiety, specific to the postpartum period. Factor 1 (Maternal Competence and Attachment Anxieties) contains 15-items which address anxieties relating to maternal self-efficacy, parenting competence, and the mother-infant relationship. Factor 2 (Infant Safety and Welfare Anxieties) has 11-items which relate to fears about infant illnesses, accidents, and cot death. Factor 3 (Practical Infant Care Anxieties) includes 7-items covering anxieties which are specific to infant care such as feeding, sleeping, and general routine. Finally, Factor 4 (Psychosocial Adjustment to Motherhood) contains 18-items which address adjustment concerns since the birth of the baby about management of personal appearance, relationships and support, work and finances, and sleep. Each answer is given a score of between 1 and 4 with the maximum score being a total of 204. Initial validation of the English-language version demonstrated a score of 112 or above may be indicative of a clinical level of anxiety [19].

The predictive validity of the measure has been examined and confirmed in relation to infant feeding outcomes and behaviours [20], and maternal bonding behaviours [21]. Across both of these studies, the PSAS demonstrated stronger predictive power than a general non-childbearing measure of anxiety.

**Fig. 1:**
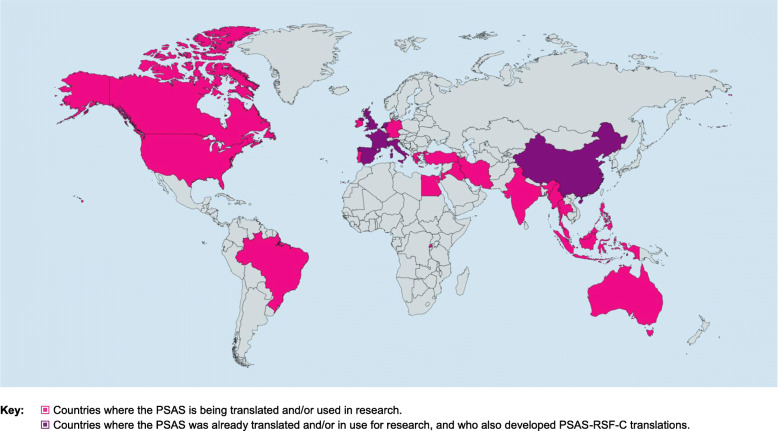
PSAS Reach. Key**:**  Countries where the PSAS is being translated and/or used in research.  Countries where the PSAS was already translated and/or in use for research, and who also developed PSAS-RSF-C translations

To date, initial validity and reliability has been demonstrated in one large UK sample [19], and more recently two Turkish samples [22, 23]. The English-language PSAS is currently being used throughout the UK, Canada, Australia, Ireland, Rwanda, and the USA. Translation of the PSAS has taken place in Italy, France, China, Spain, and The Netherlands, but are, as yet, unpublished. Further translations are currently ongoing in Brazil, Egypt, Germany, Greece, India, Indonesia, Iran, Iraq, Jordan, Malaysia, Portugal, and The Philippines. A further translation into Burmese (the language of Myanmar) is being undertaken by a research team in Thailand. (See Fig. 1).

### Study rationale

Research to understand the psychological impact of COVID-19 in perinatal populations is critical in mitigating the severity of the outbreak. Rapid progress in addressing this pandemic depends upon a coherent and integrated response from researchers [24]. There have also been global calls for the mental health sciences to work in a multi-disciplinary fashion to address the possible mental health crisis which may follow the physical health pandemic [25], and where possible make addressing mental health needs an integral part of the COVID-19 response [26]. The 51-items in the PSAS take approximately 10 min for mothers to complete which, when integrated into a survey containing a battery of psychometric scales, may be burdensome, especially during the current pandemic, where specific populations may be over-sampled. Therefore, during times of crises such as the current COVID-19 global pandemic, it is desirable to use shortened measures, to reduce participant burden. Furthermore, to the authors’ knowledge, there have been no psychometric scales (including measures of perinatal mental health) validated for use during the current pandemic.

In the UK, NICE guidelines recommend psychometric measures should contain fewer than 12 items for optimal accessibility [11]. This article reports the development of a 12-item research short-form of the PSAS, validated for use in global crises [PSAS-RSF-C]. The validation of the PSAS-RSF-C, in English, acts as a nested psychometric study within a larger on-line UK survey: PRegnancy and Motherhood during COVID-19 [The PRaM Study]. As the 51-item PSAS is currently undergoing multiple translations (all at various stages of validation), we also present, within this paper, the same 12 items, in five other languages (Italian, French, Chinese, Spanish, and Dutch). By increasing the accessibility of the PSAS, validated for use during COVID-19, we aim to enable researchers the opportunity to measure maternal anxiety, rapidly and accurately, at times of global crisis.

## Methods

### Participants

A UK sample of mothers (*N* = 710) with infants aged between birth and 12 weeks were recruited to complete an on-line survey. All data were collected from participants during the period of time in which the UK Government implemented the initial form of social ‘lockdown’ (23 March 2020–10 May 2020).

Maternal- and infant-related demographic questions were asked at the beginning of the survey (Table 1). Specific questions were also asked on the incidence of COVID-19 in the mother and any family members. Maternal age ranged between 18 and 46 years (M = 31.69, SD = 5.15) and infant age ranged between birth and 12 weeks (M = 7.92, SD = 3.67). Women were predominantly white (95%), married (57%), university educated (64%), and professionals (52%). In addition, 140 women had a clinical diagnosis of anxiety (20%); 85 had a clinical diagnosis of depression (12%); and 28 had a clinical diagnosis of PTSD (4%). Forty-nine women believed they had COVID-19 (7%), with two of these women having been tested. Additionally, 125 women believed a family member had COVID-19 (18%), with ten of these women reporting their family member had been tested. Finally, 242 women believed their birth experience had been affected by UK Government ‘lockdown’ restrictions (34%).

**Table 1:** Maternal, Infant, and COVID-19 Characteristics (*N* = 710 Mothers)

*Maternal age (mean years ± SD)*	31.69 (5.15)	**Maternal Diagnoses**	**Value n (%)**		
*Infant age (mean weeks ± SD)*	7.92 (3.67)	*Current Diagnosis of Anxiety*		*Mode of delivery*	
		Yes	140 (19.7)	Vaginal (without medical intervention)	359 (50.6)
**Maternal Characteristic**	**Value n (%)**	No	567 (79.9)	Elective caesarean section	134 (18.9)
*Ethnicity*		Prefer not to say	3 (0.4)	Emergency caesarean section	136 (19.2)
White	676 (95.2)			Vaginal birth (assisted delivery)	81 (11.4)
Pakistani	2 (0.3)	*Current Diagnosis of Depression*			
Black African	2 (0.3)	Yes	85 (12.0)	*Multiple birth*	
Black Caribbean	1 (0.1)	No	621 (87.5)	Yes	9 (1.3)
Chinese	3 (0.4)	Prefer not to say	4 (0.6)	No	701 (98.7)
Indian	7 (1.0)				
Black Other	1 (0.1)	*Current Diagnosis of PTSD*		*Infant medical complications since birth*	
Other or prefer not to say	18 (2.5)	Yes	28 (3.9)	Yes	161 (22.7)
		No	676 (95.2)	No	546 (76.9)
*Marital Status*		Prefer not to say	6 (0.8)		
Married or Co-habiting	667 (93.9)			**COVID-19 Characteristic**	**Value n (%)**
Single, Separated/Divorced, or Widowed	43 (6.1)	**Postpartum Anxiety (PPA)**	**Value n (%)**	*Suspected COVID-19*	
		*Overall PPA PSAS-RSF-C Mean (± SD)*	24.84 (6.28)	Yes	49 (6.9)
*Occupation*^a^				No	660 (93.0)
In paid employment	633 (89.2)	*Feeling that PPA has been affected by COVID-19*		Prefer not to say	1 (0.1)
Not in paid employment	77 (10.8)	Yes	438 (61.7)		
		No	267 (37.6)	*Tested for COVID-19*	
*Education Attainment*		Prefer not to say	5 (0.7)	Yes	2 (0.3)
University-level education	455 (64.1)			No	47 (6.6)
School-level education	230 (32.4)	*PPA affected by COVID-19 Mean (± SD)*^b^	7.33 (1.82)	Not Applicable	661 (93.1)
No qualifications	10 (1.4)				
Other qualification	15 (2.1)	*Clinical anxiety diagnosis overall PSAS-RSF-C Mean (± SD)*		*Family member suspected COVID-19*	
		Yes	28.35 (7.45)	Yes	125 (17.6)
*Living Status*		No	23.90 (5.60)	No	584 (82.3)
Own property	453 (63.8)			Prefer not to say	1 (0.1)
Rent – privately (from private landlord)	147 (20.7)	**Infant Characteristics**	**Value n (%)**		
Rent - local authority (state-owned housing)	63 (8.9)	*Timing of birth*		*Family member tested for COVID-19*	
Live with parents	36 (5.1)	Premature (< 37 weeks)	54 (7.4)	Yes	10 (1.4)
Other	11 (1.5)	Term (≥37 to ≤42 weeks)	652 (91.8)	No	115 (16.2)
		Post Term (> 42 weeks)	4 (0.6)	Not applicable	585 (82.4)
*Household Size (incl. participant)*					
2 people	39 (5.5)	*Birth order*		*Birth experience affected by UK Government ‘lockdown’ restrictions*	
3 people	296 (41.7)	1st	351 (49.4)	Yes	242 (34.1)
4 people	260 (36.6)	2nd	265 (37.3)	No	464 (65.4)
5 or more people	115 (16.2)	3rd and after	93 (13.1)	Prefer not to say	4 (0.6)
		Prefer not to say	1 (0.1)		

^a^Participants provided answers to the question: ‘What is your occupation? (If you are currently on maternity leave, what was your occupation prior to this)’

^b^Women who answered ‘Yes’ to their feelings of PPA being affected by COVID-19, were then asked to rate on a scale of 1–10 (whereby 1 = much less anxious to 10 = much more anxious) of how much they felt their feelings had been affected

### Design and procedure

Participants were recruited through on-line social media platforms via an advertisement providing a link to the Qualtrics survey platform. Prior to the main survey, an electronic information sheet and consent form were provided with a tick box to confirm consent. Upon completion of the survey, participants were provided with a full electronic debrief with signposting to relevant support information, and were entered into a £25 prize draw.

### Instruments

#### Postpartum Specific Anxiety Scale – Research Short Form – for global Crises [PSAS-RSF-C]

The original principal component analysis (PCA) provides factor loadings showing the strength of the relationship between the underlying PSAS factors and individual items [19]. Researchers commonly use factor loading as a scale reduction technique, preserving items with the highest factor loading [27, 28]. In-line with NICE guidelines [11], 12-items from the PSAS were selected on the basis of a review of their factor loading. The three items with the highest factor loadings (all > 0.50), were selected from each sub-scale. In the original validation of the PSAS [19], the third and fourth items from Factor 1 possessed identical factor loadings (0.66). In-line with guidelines for psychometric scale development [28, 29], Item 3 was selected over Item 4 for the PSAS-RSF-C, as it provided Factor 1 with a more comprehensive assessment of Maternal Competence and Attachment Anxieties. The final 12-items of the English-language PSAS-RSF-C can be found in Table 2.

**Table 2:** The English Language PSAS-RSF-C with Five Translations

Item Number	English Version [PSAS-RSF-C]	Italian Version [PSAS-IT-RSF-C]	French Version [PSAS-FR-RSF-C]	Spanish Version [PSAS-ES-RSF-C]	Chinese Version [PSAS-CN-RSF-C]	Dutch Version [PSAS-NL-RSF-C]
^a^1.	I have worried more about my relationship with my partner than before my baby was born.	Mi sono preoccupata di più del rapporto con il mio partner rispetto a prima che il mio bambino nascesse.	Je me suis davantage inquiétée de ma relation avec mon partenaire qu’avant la naissance de mon bébé.	Me ha preocupado más mi relación con mi pareja que antes de que naciera el bebé.	我比宝宝出生之前更担心我与伴侣的关系	Ik heb me meer zorgen gemaakt over mijn relatie met mijn partner dan voordat mijn baby was geboren.
2.	I have worried about my baby’s weight.	Mi sono preoccupata per il peso del mio bambino.	Je me suis inquiétée du poids de mon bébé.	Me preocupa el peso de mi bebé.	我担心宝宝的体重	Ik heb me zorgen gemaakt over het gewicht van mijn baby.
3.	I have worried about getting my baby into a routine.	Mi sono preoccupata di far avere al mio bambino una sua routine.	Je me suis inquiétée d’arriver à instaurer une routine avec mon bébé.	Me ha preocupado crear una rutina para mi bebé.	我为让宝宝养成习惯而发愁	Ik heb me zorgen gemaakt over het krijgen van een routine voor mijn baby.
4.	I have worried about my baby being accidentally harmed by someone or something.	Mi sono preoccupata che qualcuno o qualcosa possa nuocere accidentalmente al mio bambino.	J’ai craint que mon bébé puisse être accidentellement blessé par quelqu’un ou quelque chose.	Me preocupa que alguien o algo, por accidente, haga daño a mi bebé.	我担心宝宝会受到某人或某物的意外伤害	Ik heb me zorgen gemaakt dat mijn baby per ongeluk bezeerd raakt door iets of iemand anders.
5.	I have felt unconfident or incapable of meeting my baby’s basic care needs.	Mi sono sentita insicura o incapace di soddisfare i bisogni primari del mio bambino.	Je me suis sentie pas assez en confiance ou incapable de répondre aux besoins fondamentaux de mon bébé.	Me he sentido insegura o incapaz de satisfacer las necesidades básicas de mi bebé.	我感觉我没有信心或能力满足宝宝的基本护理需求	Ik heb me onzeker of onbekwaam gevoeld om aan de basisbehoeften van mijn baby te voldoen.
6.	I have had negative thoughts about my relationship with my baby.	Ho avuto pensieri negativi rispetto alla relazione con il mio bambino.	J’ai eu des pensées négatives concernant ma relation avec mon bébé.	He tenido pensamientos negativos sobre mi relación con mi bebé.	我对我与宝宝的关系有过消极的想法	Ik heb negatieve gedachten gehad over de relatie die ik met mijn baby heb.
7.	I have worried about my baby’s milk intake.	Mi sono preoccupata per la quantità di latte assunta dal mio bambino.	Je me suis inquiétée de la quantité de lait prise par mon bébé.	Me preocupa la cantidad de leche que toma mi bebé.	我担心宝宝的牛奶摄入量	Ik heb me zorgen gemaakt over de hoeveelheid melk die mijn baby drinkt.
8.	I have worried that my baby will stop breathing while sleeping.	Ho avuto paura che il mio bambino smettesse di respirare durante il sonno.	J’ai craint que mon bébé ne cesse de respirer pendant son sommeil.	Me preocupa que mi bebé deje de respirar mientras duerme.	我担心宝宝睡觉时会停止呼吸	Ik heb me zorgen gemaakt dat mijn baby zal stoppen met ademen tijdens het slapen.
9.	I have felt that my baby would be better cared for by someone else.	Ho pensato che il mio bambino sarebbe meglio accudito da qualcun altro.	J’ai senti que quelqu’un d’autre prendrait mieux soin de mon bébé.	He sentido que mi bebé estaría mejor cuidado por otra persona.	我感觉我的宝宝最好由别人来照顾	Ik heb het gevoel gehad dat mijn baby beter zou worden verzorgd door iemand anders.
^a^10.	I have felt resentment towards my partner.	Ho provato risentimento nei confronti del mio partner.	J’ai eu du ressentiment envers mon partenaire.	He sentido resentimiento hacia mi pareja.	我对我的伴侣感到怨恨	Ik heb gevoelens van wrok gehad naar mijn partner.
11.	I have repeatedly checked on my sleeping baby.	Ho ripetutamente controllato il mio bambino mentre dormiva.	Je suis allée surveiller de façon répétée mon bébé pendant son sommeil.	He comprobado repetidas veces el estado de mi bebé mientras dormía.	宝宝睡觉时, 我会反复查看他/她	Ik heb herhaaldelijk mijn slapende baby gecontroleerd.
12.	I have felt tired even after a good amount of rest.	Mi sono sentita stanca anche dopo una buona quantità di riposo.	Je me suis sentie fatiguée même après beaucoup de repos.	Me he sentido cansada incluso después de un buen descanso.	即使得到充分休息, 我仍感觉很累	Ik heb me moe gevoeld, zelfs na een goede hoeveelheid rust.

^a^Indicates items which may not be applicable to all mothers’ circumstances and therefore can be left blank by the participant

#### Edinburgh Postnatal Depression Scale [EPDS]

The EPDS [30] is a 10-item self-report questionnaire which screens for postnatal depressive symptomatology. It is commonly utilised and recommended screening scale for postnatal depression. Scored out of 30, higher levels of postpartum depressive symptoms are indicated by high scores on the scale, with a score of greater than 10 indicative of a probable postpartum depression. Items three, four, and five cluster on an anxiety factor [EPDS-3A] to indicate postpartum anxiety [31, 32]. Scored out of nine, with scores of six or above indicating probable postpartum anxiety.

#### State-Trait Anxiety Inventory – State scale [STAI-S]

The STAI-S is a sub-scale of the STAI [33]. It is a 20-item self-report questionnaire which measures situational anxiety. The STAI-S is a valid and reliable measure used frequently in perinatal samples of women [18]. It is scored out of 80, on a four point Likert scale, with higher scores indicating higher levels of anxiety.

#### Parenting Sense of Competence scale [PSoC]

The PSoC is a frequently used measure of parenting competence, with seven items and two sub-scales [34]. Each item is rated on a six-point Likert scale with higher scores indicating a higher sense of parenting competence.

### Method of analysis

Data taken from The UK PRaM Study (*N* = 710) were randomly split into two samples: One for the exploratory factor analysis (*n* = 344), the other for the confirmatory factor analysis (*n* = 366).

#### Exploratory factor analysis (group 1; n = 344)

Due to these data being ordinal (as scored on a four-point Likert scale), a parallel analysis was conducted using the simulated polychoric correlation matrix in order to identify the number of likely components in the data. Following this, a PCA with oblique rotation (oblimin) was conducted, again using the polychoric correlation matrix. Notably, results were identical when the raw data were analysed.

#### Confirmatory factor analysis (group 2; n = 366)

A confirmatory factor analysis was performed using MPLUS version 8.4 [35], using robust unweighted least squares estimation [36]. Items were free to load onto their corresponding latent factors, and latent factors were free to correlate with each other. Model fit was assessed using the Comparative Fit Index [CFI] and the Tucker-Lewis Index [TLI], where values of above .90 are deemed ‘acceptable’, and values above .95 are deemed ‘good’. The Root Mean Square Error of Approximation (RMSEA) [37] indicates a good fit (<.05), a fair fit (.05 <> .08), a mediocre fit (.08 <> .10), and a poor fit (>.10). The Standardized Root Mean Square Residual [SRMR] is considered a good fit where values are less than .08 [38]. Modification indices were also inspected, and if in excess of 20, covariance pathways were added between error terms (if conceptually appropriate, items loaded onto the same factor).

#### Internal consistency

Internal reliability of the full scale and each subscale was estimated by computing McDonald’s ω through the polychoric correlation matrix. This was computed for both data sets.

#### Convergent validity

Correlation analyses were performed to examine the associations between the PSAS-RSF-C and theoretically related measures of anxiety (i.e. EPDS-A; STAI-S), depression (i.e. EPDS), and parenting competence (i.e. PSoC).

#### Preliminary screening accuracy

A receiver operating characteristic [ROC] analysis was undertaken to distinguish between those with and without a self-reported current clinical diagnosis of anxiety.

## Results

### Factor structure of the PSAS-RSF-C

The factor structure of the PSAS-RSF-C (Table 3) was examined using data from all the participants in Group 1 (*n* = 344). The parallel analysis suggested there were four factors which are consistent with the original 51-item measure. Sampling adequacy for the 12-item scale was excellent (Kaiser-Meyer-Olkin; KMO = 0.80) and Bartlett’s test of sphericity demonstrated correlations between items were large enough for PCA (*χ*^2^ (66) = 284.17, *p* < .001). The PCA revealed four factors, which in combination explained 75% of the total variance. The UK-based PSAS Working Group [VF, SAS, JAH, JCGH, SMD, PaCh] conducted a theoretical review of the factor loadings after oblique (direct oblimin) rotation, which revealed that the factor structure of the PSAS-RSF-C was identical in nature to that of the original PSAS. Three items loaded onto each of the four factors which they belonged to in the long form: Maternal Competence and Attachment Anxieties; Infant Safety and Welfare Anxieties; Practical Infant Care Anxieties; and Psychosocial Adjustment to Motherhood. The four factors had good reliability, with McDonald’s ω ranging from .74 to .88 (see Table 3). Furthermore, the overall scale had good reliability (McDonald’s ω = .87).

**Table 3:** Factor structure of the PSAS-RSF-C

	Rotated components			
Scale item	1	2	3	4
*Factor 1: Maternal Competence and Attachment Anxieties*				
1. I have had negative thoughts about the relationship with my baby	**0.85**	−0.02	−0.01	0.13
2.I have felt that my baby would be better cared for by someone else	**0.85**	0.04	0.06	0.04
3.I have felt unconfident or incapable of meeting my baby’s basic care needs	**0.76**	0.03	0.25	0.01
*Factor 2: Infant Safety and Welfare Anxieties*				
4. I have worried about my baby being accidentally harmed by someone or something else	0.40	**0.69**	−0.06	−0.05
5. I have repeatedly checked on my sleeping baby	−0.26	**0.86**	0.14	0.08
6. I have worried that my baby will stop breathing while sleeping	0.09	**0.91**	−0.03	0.00
*Factor 3: Practical Infant Care Anxieties*				
7. I have worried about my baby’s milk intake	0.11	0.06	**0.86**	−0.03
8. I have worried about my baby’s weight	0.00	0.00	**0.92**	0.01
9. I have worried about getting my baby into a routine	0.28	−0.04	**0.51**	0.19
*Factor 4: Psychosocial Adjustment to Motherhood*				
10. I have felt resentment towards my partner	0.17	0.04	−0.19	**0.80**
11. I have felt tired even after a good amount of rest	−0.16	0.11	0.21	**0.68**
12. I have worried more about my relationship with my partner than before my baby was born	0.03	−0.05	0.06	**0.84**
% of variance explained	22	18	18	17
McDonald’s Omega	.88	.83	.82	.74

All significant loadings in bold

### Confirmation of factor structure

**Fig. 2:**
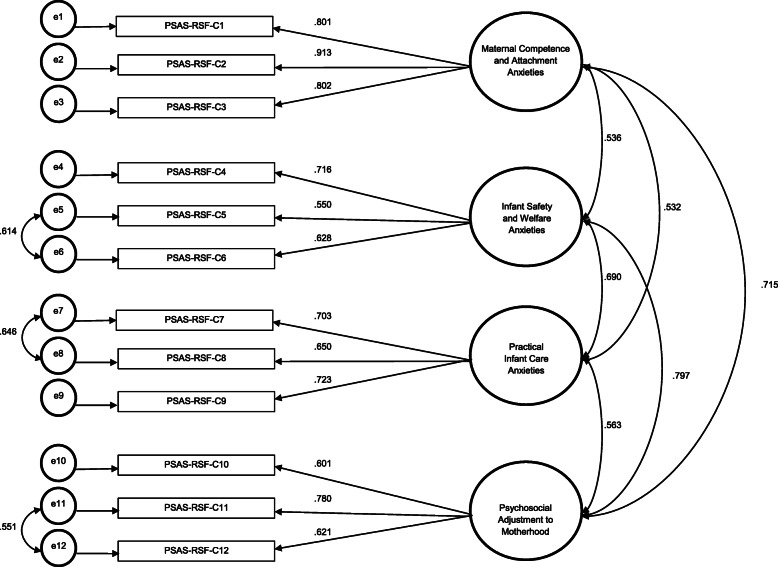
Standardised Factor Loadings

The initial model was a moderate to good fit of the data (CFI = .928, TLI = .901, RMSEA = .096, SRMR = .067). Modification indices indicated a covariance should be added between three pairs of residuals (Fig. 2). As a result, the model fit improved (CFI = .973, TLI = .960, RMSEA =. 055, SRMR = .045). All items significantly loaded onto each factor (*p* < .001; see Fig. 2 for the standardised factor loadings). The overall scale retained good reliability (McDonald’s ω = .87).

### Convergent validity

The participants who completed all convergent measures were included in these analyses. The PSAS-RSF-C total score was significantly correlated with theoretically related measures of anxiety (i.e. EPDS-A [*n* = 710]; STAI-S [*n* = 709]), depression (i.e. EPDS [*n* = 710]), and parenting competence (i.e. PSoC [*n* = 666]) indicating good convergent validity (Table 4).

**Table 4:** Pearson product-moment correlations demonstrating convergent validity between the PSAS-RSF-C and other validated measures of maternal mental health

	EPDS-A	STAI-S	EPDS	PSoC
PSAS-RSF-C	.57*	.62*	0.67*	−0.54*

*p < .001 (one-tailed)

### Preliminary screening accuracy of PSAS-RSF-C

**Fig. 3:**
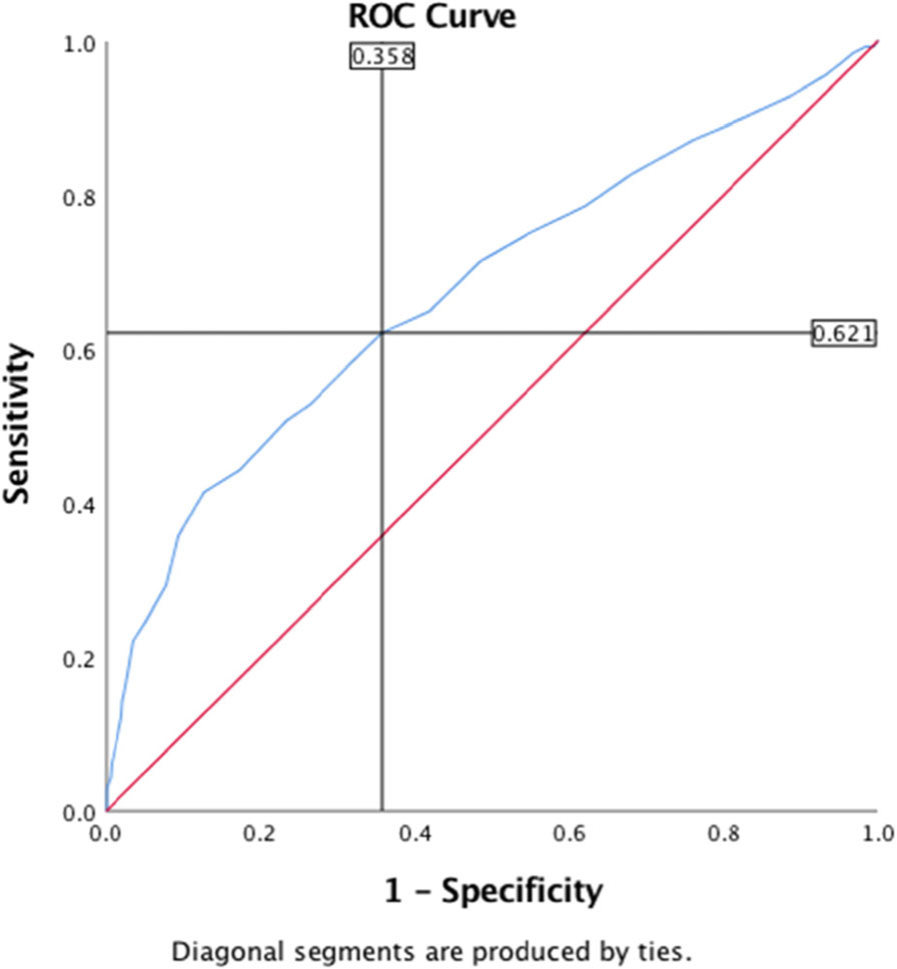
Receiver Operating Characteristic [ROC] curve analysis. Area under the curve = 0.68

First, An independent samples t-test demonstrated that the mean PSAS-RSF-C scores for those with a self-reported clinical diagnosis of anxiety (*n* = 140; M = 28.35; SD = 7.45) were significantly higher than those without a self-reported clinical diagnosis (*n* = 567; M = 23.93; SD = 5.60) t (705) = − 7.79; *p* < .001. Then, to preliminarily evaluate the performance of the PSAS-RSF-C in distinguishing between those with or without a current clinical diagnosis of anxiety, a Receiver Operating Characteristic [ROC] analysis was conducted. A statistically significant ROC curve (AUC = .68; SE = .03; p < .001; 95% CI .62 to .73) revealed the optimal cut-off PSAS-RSF-C score for detecting clinical levels of anxiety was 26 out of a total of 48 with a sensitivity and specificity of .62 and .64, respectively (Fig. 3).

### International use of the PSAS-RSF-C

Translation of the PSAS follows traditional methods of psychometric scale translation [39–42]. In brief, this requires at least three researchers to supply separate versions of the PSAS into the desired language. These translations are then given to an independent back-translator [39], who is unfamiliar with the PSAS. The back-translator will select the most eloquent translation of each item from the three translated versions [40]. This single back-translation is then checked with the UK-based PSAS Working Group, who checks the back-translation against the original PSAS for inconsistencies and intended meaning [41]. The items which make up this final back-translated version are the items which will be used for the final translated version. Inconsistencies at any stage of the translation process are discussed amongst the translating team, with irreconcilable discrepancies being referred to the UK-based PSAS Working Group [42].

We therefore have selected the same 12-items in the currently unvalidated, but translated Italian, French, Spanish, Chinese, and Dutch versions of the 51-item PSAS to form a PSAS-RSF-C for each country (PSAS-IT-RSF-C, PSAS-FR-RSF-C, PSAS-CN-RSF-C, PSAS-ES-RSF-C, and PSAS-NL-RSF-C, respectively, Table 2).

## Discussion

The primary aim of the current study was to develop a brief and accessible measure of postpartum anxiety. We aimed to validate the tool for research use in global crises such as the current pandemic, and further explore its scope for clinical usage. To that end, we developed a 12-item short-form of the English-language PSAS and examined the psychometric properties and diagnostic accuracy of the measure in two samples of women during the UK ‘lockdown’.

The selection of items for the short-form was informed by the aim of preserving the originally proposed four factor structure of the PSAS [19] with domains of both maternal- and infant-focused anxiety. This was in order to maintain breadth of content coverage, in-line with psychometric guidance [27–29]. The validation of the English-language PSAS-RSF-C shows promising psychometric properties which would be useful for rapid measurement of maternal anxiety in the current global crisis, and any crises which may occur in the future.

The current findings demonstrate the robustness of the PSAS across diverse psycho-social contexts. Exploratory factor analyses in one sample demonstrated a simple four-factor structure, identical to the original 51-item version [19], expressed as (1) Competence and Attachment Anxieties; (2) Infant Safety and Welfare Anxieties; (3) Practical Baby Care Anxieties; and (4) Psychosocial Adjustment to Motherhood. Confirmatory factor analyses in a second sample demonstrated an excellent fit of the measurement model. The overall PSAS-RSF-C and its sub-scales demonstrated good reliability in both samples. This suggests the types of anxieties new mothers are experiencing during the current crisis are comparable to those which occurred pre-pandemic, and are maternal- and infant-focused in nature. This indicates a continued critical need to further understand the experience and impact of perinatal anxiety during the current crisis [3, 43–45].

The diagnostic accuracy of the original 51-item PSAS to detect individuals with self-reported diagnosis of anxiety was examined by means of ROC analyses [19]. To our knowledge, there are no other measures of anxiety validated during the current global COVID-19 pandemic, with which to compare these findings. However, when compared to the original 51-item PSAS, the results of this study indicate a good and comparably good diagnostic accuracy for the PSAS-RSF-C. We envisage the PSAS-RSF-C can be applied to a clinical setting to assist healthcare professionals in identifying mothers with problematic anxiety, as part of a broader clinical assessment. Therefore, we propose a cut-off score of 26 which provides a good balance between the sensitivity and specificity of the tool [46]. However, we recognise mental health may be elevated during the current pandemic [3–6, 8, 9, 26], and as such, clinical judgement should be used in parallel with PSAS-RSF-C scores.

The translated versions of the PSAS-RSF-C into five global languages (Italian, French, Chinese, Spanish, and Dutch) will enable a broad use of the tool in order for researchers and clinicians globally to assess maternal mental health when undertaking rapid response research and clinical assessments during the pandemic. Whilst each translation requires subsequent validation, work is already underway to do so. This will allow for a more comprehensive assessment of global maternal mental health during the COVID-19 pandemic, as well as providing the opportunity to compare between datasets utilising the same scale items, in different languages.

### Strengths, limitations, and future directions

A major strength of this study is that, to the authors’ knowledge, this is the first postpartum-specific psychometric scale to be validated for use during the COVID-19 global pandemic, and in being so, is also one of the first perinatal psychometric tools to be validated in the current crisis, with others including the Pandemic-Related Pregnancy Stress Scale [47]. This means postpartum anxiety can be effectively measured during this pandemic, and similar global crises in the future. Furthermore, many psychometric studies inappropriately use factor analyses developed for interval-level data, when the psychological construct, and the measurement of it, is, in fact, ordinal in nature. A strength of this study is the use of a polychoric correlation matrix, which overcomes this common, but often statistically inappropriate practice [48]. Additionally, we report McDonald’s Omega, to appraise reliability, as opposed to Cronbach’s Alpha, which focuses on the greatest lower bounds estimate [49]. Finally, providing a validated 12-item research short-form of the PSAS means it can be more readily adopted into studies containing a large battery of tests during COVID-19 and other such global crises, without being prohibitively long. Whilst the diagnostic accuracy was found to be good in this validation study, we recommend erring on the side of caution when making clinical decisions based on this research short-form alone.

A limitation is the use of an on-line convenience sample. This recruitment, whilst pragmatic for the rapid response nature of this research, lacked sampling control. The sample were predominantly white, married women, with university education and professional occupations. There was also a high proportion of assisted vaginal births and caesarean sections, and whilst the cause of this remains unknown, it has been suggested women have delayed seeking care during the pandemic, leading to higher incidence rates of obstetric complications [50]. The psychometric properties of the PSAS-RSF-C may, therefore, vary in other populations and it should be subject to replication studies using diverse samples.

Future research efforts should be directed towards the global validation of the PSAS and the PSAS-RSF-C. Whilst the five translations of the PSAS-RSF-C presented in this paper have not yet been subjected to validation studies, validation work must be conducted to ensure the validity and reliability of both the 12-item PSAS-RSF-C and the 51-item PSAS in other languages.

Previous work examining the predictive validity of the PSAS [20, 22] consistently finds it is a more powerful predictor of perinatal outcomes than a general measure (e.g. STAI [33]). Consequently, this tool would be useful in longitudinal studies aiming to better understand the persistent mental health impact of the pandemic (and other such global crises in the future) on maternal and infant outcomes. Preliminary work demonstrates the sub-scales of the PSAS have differential effects on maternal and infant outcomes [51]. Further investigations of the sub-scales could provide greater level of detail in terms of identifying specific risk factors and mechanisms of PPA and may offer opportunities for targeted intervention.

## Conclusion

Following the calls for mental health to be addressed during and after the global crisis, the PSAS-RSF-C offers one way in which to reliably measure maternal mental health in the postpartum period. To the best of the authors’ knowledge, this is the first postpartum-specific psychometric scale to be validated for use during the COVID-19 pandemic, and in being so, is also one of the first perinatal psychometric tools to be validated during this current crisis. In doing so, we provide opportunity for researchers and clinicians to measure postpartum anxiety accurately, whilst laying foundations for further global psychometric work to be undertaken during the current crisis, and in those which will present in the future.

## Data Availability

The datasets used and/or analysed during the current study are available from the corresponding author on reasonable request.

## Abbreviations

EPDS: Edinburgh Postnatal Depression Scale
NCCMH: National Collaborating Centre for Mental Health
NICE: National Institute of Health and Care Excellence
PCoS: Parenting sense of competence scale
PPA: Postpartum anxiety
PSAS: Postpartum Specific Anxiety Scale
PSAS-RSF-C: Postpartum Specific Anxiety Scale – Research Short Form – for global Crises.
RCOG: Royal College of Obstetricians and Gynaecologists
SARS-CoV-2: Severe Acute Respiratory Syndrome Coronavirus 2 (a.k.a. COVID-19)
STAI: State-Trait Anxiety Inventory
UKRI: UK Research and Innovation
WHO: World Health Organization
